# Pharmacogenomic Testing and Antithrombotic Therapy: Ready for Prime Time?

**DOI:** 10.5041/RMMJ.10105

**Published:** 2013-01-30

**Authors:** David R. Holmes

**Affiliations:** Past President, American College of Cardiology (ACC); Cardiovascular Diseases, Mayo Clinic, Rochester, MN, USA

**Keywords:** Antiplatelet therapy, clopidogrel, pharmacogenomics, stent thrombosis

## Abstract

Pharmacogenomics is the study of an individual’s interaction with a specific drug based upon the genetic make-up of the individual. Pharmacogenomic testing can be a powerful tool in testing a drug’s potential efficacy and toxicity on an individual patient. For this tool to be used correctly, certain criteria have to be met. First and foremost is the strength of association between the genetic variation and the drug’s interaction. The predictiveness of pharmacogenomics for the individual patient must be factored in as well. If these criteria are not met, requiring pharmacogenomic testing is at best a waste of money and in some cases can endanger the patient’s life. Stent thrombosis is a serious and many times fatal outcome in a small minority of patients who have received drug-eluting stents. Here, we discuss a case in which the FDA issued a “boxed warning” about the use of the anti-clotting medication, clopidogrel, used to prevent stent thrombosis, the pharmacogenomic data available at the time the warning was issued, and the medical community’s response to the FDA’s warning. This article also discusses developments in the field of anti-clotting therapy since the FDA’s warning.

## PHARMACOGENOMICS AND THERAPEUTICOGENOMICS

Personalized medicine aims to give individuals the best care tailored to their unique genetic make-up. Genomics, the study of an organism’s genome, has many practical medical applications. Two such applications are pharmacogenomics and therapeuticogenomics.

Pharmacogenomics studies the influence of genetic variations on the patient’s response to specific drugs, such as the correlation between the efficacy or toxicity of a certain drug and a specific gene expression or a single-nucleotide polymorphism. One concrete example involves the cytochrome P450 (CYP) family of liver enzymes. These enzymes are responsible for breaking down more than 30 different classes of drugs. DNA variations in genes that code for these enzymes can influence their ability to metabolize certain drugs.

Therapeuticogenomics deals with therapeutic modalities for diseases that have a genetic component. For certain diseases, diet and lifestyle changes (e.g. exercise and the cessation of smoking), together with medications, can alleviate the adverse outcomes of the disease. For example, in the case of genetic predisposition to hypercholesterolemia, once the genetic predisposition has been identified, these types of treatments can be given as prophylactic measures, even at a relatively young age. Unfortunately, this is not the case for many other diseases. One such example is breast cancer in women who have mutations in the BRCA genes. The prevalence of these mutations in the general population is roughly 1 in 800. They are responsible for up to 25% of early-onset breast malignancy and up to 90% of early-onset cancers in families with a history of breast malignancies.^1^ In this case, simple lifestyle changes might somewhat lower the chances of getting cancer, but there are no simple reversible prophylactic measures which can be taken. When such a mutation is found in a family, should all the females of that family be tested? Should they all be informed of the results? Should these women undergo enhanced surveillance? Should all women found to be positive for the mutated genes undergo prophylactic mastectomies? These questions do not have easy answers especially when we are dealing with females of all different ages.

The application of pharmacogenomics in medicine seems less problematic. People for whom certain genetic variations hinder the metabolism of a certain drug, thus making that drug either ineffective or toxic, should simply not be prescribed that specific drug. However, the real picture is slightly more complicated.

There are four criteria for judging the clinical usefulness of pharmacogenomics. Firstly, the strength of association with the clinical problem is essential. Clearly, if the strength of association is low, so is the use of pharmacogenomics. Secondly, we need to evaluate the clinical importance of the specific clinical problem to justify the use of pharmacogenomics. Trivial medical problems do not warrant the use of pharmacogenomics. Thirdly, we need to factor in the predictiveness of pharmacogenomics for the individual patient, and lastly, other available treatment options must be considered. These four factors must be taken into account when bringing pharmacogenomics into the practice of medicine.

## CARDIOVASCULAR DISEASE, LATE STENT THROMBOSIS, AND PHARMACOGENOMICS

Heart disease fits the criterion of clinical importance. More than 2,200 Americans die of cardiovascular disease (CVD) each day,^2^ and there are many pharmacogenomic implications for CVD.^3^–^5^ If a life-saving drug was shown to be less effective for people who carry a certain genetic marker, and, even more pertinent, if as a result of this genetic predisposition they were at risk if given a certain drug, it is clearly medically relevant.

**Figure 1:**
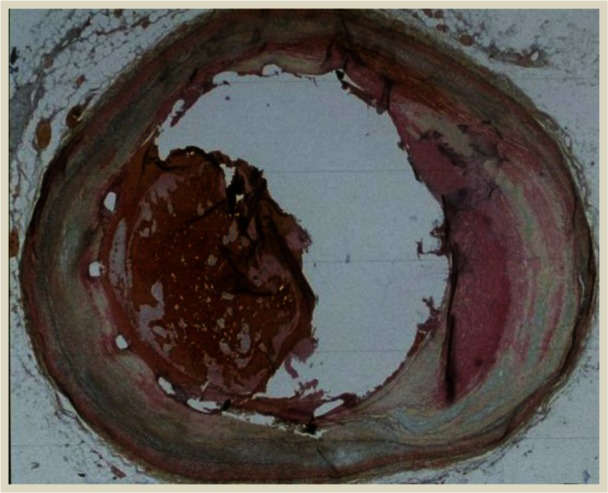
**Stent thrombosis.**

One common procedure performed on patients with acute CVD is stenting. Over 1 million stent procedures are annually performed in the United States.^6^ Although drug-eluting stents have been very successful in preventing re-narrowing, or restenosis of the coronary arteries, these stents carry a slight increase in risk for late stent thrombosis (Figure 1). The occurrence of late stent thrombosis is the result of several factors such as incomplete stent apposition. The frequency of late stent thrombosis occurrence is low, but the risk continues over time. Despite the low frequency, the clinical implication of stent thrombosis is dire since the chance of death or myocardial infarction from stent thrombosis is 40%–60%. Therefore, patients with drug-eluting stents are treated with dual antiplatelet therapy (aspirin plus clopidogrel, ticagrelor, or prasugrel) for the recommended duration.

## ANTIPLATELET THERAPY AND CLOPIDOGREL

**Figure 2:**
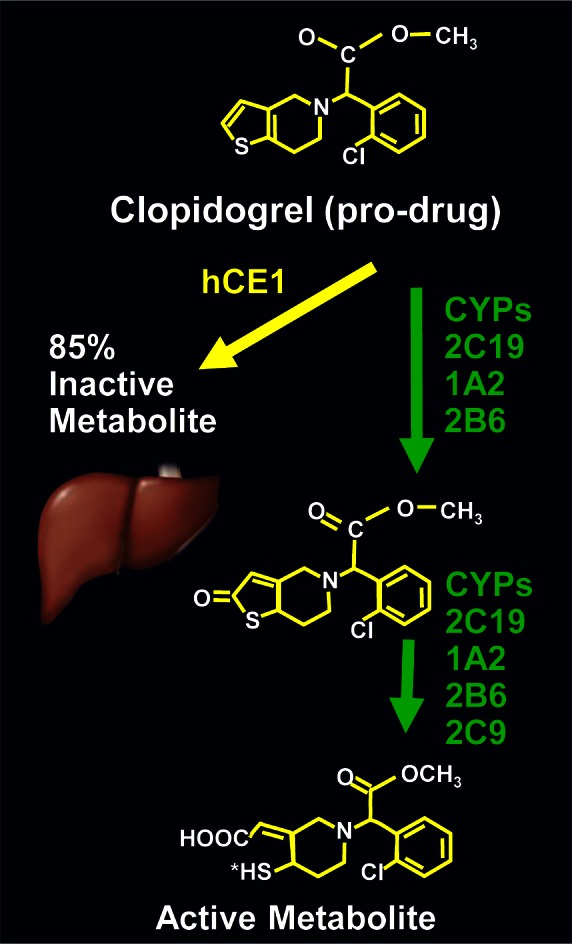
**Chemical composition of clopidogrel.**

The antiplatelet therapy drug, clopidogrel (Plavix^®^) is a prodrug which is activated in the liver in a two-step process by cytochrome P450 enzymes (Figure 2). The bioavailability of clopidogrel is determined by the genetic make-up of these enzymes and other enzymes in addition to intestinal absorption. Clopidogrel acts upon an ADP receptor that is found on platelet cell membranes. Clopidogrel specifically and irreversibly inhibits the P2Y12 subtype of the ADP receptor, thus inhibiting the activation of platelets and the platelets’ eventual cross-linking by the protein fibrin.^7^ The individual variability of ADP-induced platelet aggregation in response to clopidogrel ranges from less than 10% to almost 100% inhibition of platelet aggregation. The distribution across this range precludes the dichotomous separation into “responders” and “non-responders.”^8^

Clopidogrel is a very popular drug. It is marketed worldwide in nearly 110 countries, and for several years it was the second best-selling drug worldwide.^9^ Therefore, adverse information on such a drug will have an impact on the multitude of patients taking this drug along with their physicians and families.

## BOXED WARNING

On March 12, 2010 the Food and Drug Administration (FDA) sent out a boxed warning (also known as a “black box warning”) about clopidogrel. A boxed warning is sent out when it is discovered that side-effects of the drug may lead to death or serious injury. In these instances, the FDA requires that the manufacturers prominently place a warning on the drug’s package. The FDA warning about clopidogrel stated the following:

The U.S. Food and Drug Administration today added a boxed warning to the anti-blood clotting drug Plavix (clopidogrel), alerting patients and health care professionals that the drug can be less effective in people who cannot metabolize the drug to convert it to its active form.

Plavix reduces the risk of heart attack, unstable angina, stroke, and cardiovascular death in patients with cardiovascular disease by making platelets less likely to form blood clots. Plavix does not have its anti-platelet effects until it is metabolized into its active form by the liver enzyme, CYP2C19.

People who have reduced functioning of their CYP2C19 liver enzyme cannot effectively convert Plavix to its active form. As a result, Plavix may be less effective in altering platelet activity in those people. These “poor metabolizers” may not receive the full benefit of Plavix treatment and may remain at risk for heart attack, stroke, and cardiovascular death.

It is estimated that 2–14% of the U.S. population are poor metabolizers. The FDA recommends that health care professionals consider alternative dosing of Plavix for these patients, or consider using other anti-platelet medications. Tests are available to assess CYP2C19 genotype to determine if a patient is a poor metabolizer.

Patients should not stop taking Plavix unless told to do so by their health care professional. They should talk with their health care professional if they have any concerns about Plavix.^10^

One of the studies that the FDA relied upon showed that healthy subjects who had been given clopidogrel and were carriers of at least one *CYP2C19* reduced-function allele had a relative reduction of 32.4% in plasma exposure to the active metabolite of clopidogrel, as compared with non-carriers. Carriers also had an absolute reduction in maximal platelet aggregation in response to clopidogrel that was 9 percentage points less than that seen in non-carriers. Among clopidogrel-treated subjects in the TRITON–TIMI 38 trials, carriers had a relative increase of 53% in the composite primary efficacy outcome of the risk of death from cardiovascular causes, myocardial infarction, or stroke, as compared with non-carriers.^11^

## AHA/ACCF RESPONSE TO THE FDA WARNING

The warning sent out by the FDA coincided with the start of the annual meeting of the American College of Cardiology (ACC), where 20,000 cardiovascular professionals were gathered. All those present at the meeting received an e-mail alert from the FDA stating: “The FDA issues a boxed warning for CYP2C19-linked poor metabolism of Plavix.” Since all the physicians at the meeting had patients who were being treated with Plavix, they were immediately inundated with a barrage of e-mails from their patients, their patients’ families, and their patients’ lawyers, all concerned about this warning.

As a response to the FDA warning, a committee was immediately convened and set out to provide guidance for all the physicians who had to deal with the aftermath of the boxed warning. The committee comprised experts from the American College of Cardiology Foundation (ACCF) and the American Heart Association (AHA).

The findings of this committee were published 2 months after the FDA warning.^12^ The main findings were that there is substantial individual variability in the response to clopidogrel, which may be due to pharmacokinetic (PK) or pharmacodynamic (PD) differences. These differences are due to a number of factors such as age, body mass index, co-morbidities such as diabetes and dyslipidemia, and other unidentified factors. Genetic variability plays a role as well, but it explains only a small portion of the variability seen.

The role of genetic variability was seen in a study done on a homogenous population of healthy Amish adults (Pharmacogenomics of Antiplatelet Intervention—PAPI). In this study, a gene dose effect of CYP2C19*2 on clopidogrel reduction of ADP-induced platelet aggregation was seen. However, the genotype variability only accounted for 12% of the variability in clopidogrel response.^13^

In addition, other genetic variations may also affect the PK, PD, and clinical efficacy of clopidogrel. There are additional CYP genes such as 2C19, 2C9, 2B6, 3A4, 3A5, and 1A2. The adenosine triphosphate-binding cassette containing gene ABCB1, also known as the multidrug resistant (MDR1) gene, was shown to affect the metabolism of this drug as well. Large differences in the bioavailability of clopidogrel were seen among carriers of the wild-type gene as compared with those carrying the mutated form.^14^

Point-of-care assays for these genetic mutations were not available at the time when this article was being written. In addition, the positive predictive value of CYP2C19 loss-of-function genetic polymorphisms is estimated to be between 12% and 20% in patients who have acute coronary syndrome (ACS) and are undergoing percutaneous coronary interventions (PCIs).

The conclusion of the committee was that health care providers should adhere to existing evidence-based guidelines for clopidogrel and other antiplatelet therapies that have been published by the professional societies. Clinicians should be aware that genetic variability in response to clopidogrel may affect platelet inhibition. However, due to lack of clinical data, specific recommendations for routine genetic testing cannot be offered at this time. Careful clinical judgment should be used to assess the significance of the variability in an individual’s response to clopidogrel and its associated risk to the patient.

## INCREASING THE DOSAGE OF CLOPIDOGREL

The FDA’s major concern when issuing the boxed warning regarding the use of clopidogrel was for patients with two CYP2C19 loss-of-function alleles who were shown to be low responders to clopidogrel. The concern was that these patients did not have enough active metabolite to prevent stent thrombosis. As a result, a study was conducted, the GRAVITAS trial (Gauging Responsiveness with A VerifyNow Assay: Impact on Thrombosis and Safety), in which patients were screened based on their phenotype of blood clotting. Those who had high residual platelet reactivity were selected for the study. Half of the patients were given a double dose of clopidogrel (600-mg initial dose, 150 mg daily thereafter) for 6 months.^15^ Those who received the double dose showed reduced platelet reactivity after 6 months. However, the use of high-dose clopidogrel as compared with the standard dose of clopidogrel did not reduce the incidence of death from cardiovascular causes, non-fatal myocardial infarction, or stent thrombosis.

One possible reason for the failure of the GRAVITAS trial was the possibility that doubling the dose is not enough. A smaller study (ELEVATE–TIMI 56) was conducted in patients with stable cardiovascular disease. They were genotyped into the following groups: normal, CYP2C19*2 heterozygotes, and CYP2C19*2 homozygotes. Carriers of the loss-of-function CYP2C19*2 allele were given up to four times the daily dose of clopidogrel (300 mg/daily).^16^ The conclusion from this study was that tripling the maintenance dose of clopidogrel to 225 mg daily in CYP2C19*2 heterozygotes achieved levels of platelet reactivity similar to those seen with the standard 75-mg dose in non-carriers. However, no comparable degrees of platelet inhibition were seen in CYP2C19*2 homozygotes even when receiving the 300 mg daily dose. There were no incidences of death from cardiovascular causes, no strokes or major or minor bleeding in this trial. In conclusion, even if there was some improvement in platelet function, it did not impact clinical outcomes.

## META-ANALYSIS AND CYP2C19 GENOTYPING

In order to clarify the disparate data seen in different trials, a meta-analysis of 32 studies consisting of 42,016 patients was conducted to see whether there was statistical justification for CYP2C19 genotyping.^17^ The conclusion was as follows: “Although there was an association between the CYP2C19 genotype and clopidogrel responsiveness, overall there was no significant association of genotype with cardiovascular events.” However, this meta-analysis showed that there was a slight increase in stent thrombosis in a small group of patients who had a CYP2C19 loss-of-function allele.

Another meta-analysis was conducted with slightly different inclusion criteria. This meta-analysis included some cohort studies, retrospective studies, sub-studies, prospective case cohorts, and case control studies.^18^ The authors concluded that the gathered information from the genetic association studies did not indicate a substantial or consistent influence of CYP2C19 gene polymorphisms on the clinical efficacy of clopidogrel. Therefore, the current evidence does not support the use of individualized CYP2C19 genotyping. They did not even find a weak signal of elevated stent thrombosis in patients who had a CYP2C19 loss-of-function allele that was seen in the first meta-analysis.

## NEW ANTI-CLOTTING THERAPY DRUGS

The whole controversy about genetic testing is due to the fact that clopidogrel needs to be metabolized to become an active drug. However, new agents such as prasugrel, ticagrelor, and elinogrel do not undergo CYP2C19 metabolism. They are active, or almost active, drugs, and genetic variants do not appear to affect their metabolism. Therefore, instead of genotyping, we should prescribe a drug that works on all patients regardless of their genotype.

The efficacy of these new drugs (prasugrel, ticagrelor, and elinogrel) as compared to clopidogrel was shown in a number of studies. One such study compared the efficacy of clopidogrel and prasugrel. The TRITON–TIMI 38 trial had 2,932 patients who were genotyped for the CYP2C19 and ABCB1 genes. Roughly half of the patients were treated with clopidogrel and the other half with prasugrel. The trial period was 15 months.^19^ When the genetic components of the patients were analyzed, it was found that when both ABCB1 and CYP2C19 are mutated, there is indeed a risk for major adverse events for patients who carry a double mutation and receive clopidogrel. This effect was not seen on patients who received prasugrel.

**Figure 3:**
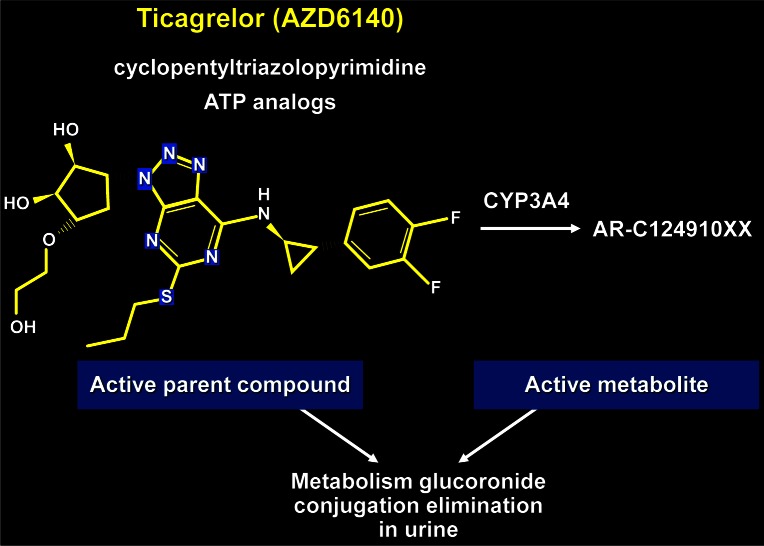
**Chemical composition of ticagrelor.**

Ticagrelor (Figure 3) is an active drug that does not have to be metabolized. A trial was conducted in which ticagrelor was compared to clopidogrel (the PLATO trial).^20^ A total of 10,285 patients with acute coronary syndrome were genotyped for CYP2C19 and ABCB1 and then randomized to receive ticagrelor or clopidogrel. Ticagrelor was found to be more efficacious for acute coronary syndrome than clopidogrel, irrespective of CYP2C19 and ABCB1 polymorphisms. The researchers concluded that the “use of ticagrelor instead of clopidogrel eliminates the need for presently recommended genetic testing before dual antiplatelet treatment.”

The European Society of Cardiology (ESC) published guidelines reflecting these studies (Table 1).^21^ They recommend that ticagrelor be given as the initial treatment and be given even to patients who had been previously treated with clopidogrel. The rationale behind this recommendation is that ticagrelor obviates the necessity for genetic testing and therefore should be the front-line drug.

## CONCLUSION

There is still great room for clinical judgment in this field. Clinicians must decide which drug to give. If clopidogrel is given, should the dose be doubled or tripled? Should the patients be initially tested, and what sort of test should be done, genotyping or phenotyping? Should these newer drugs be given only during the first month after a stent is inserted and then clopidogrel since the first month is when most cases of stent thrombosis occur, or should the patient be given these newer drugs indefinitely? Since clopidogrel will soon be taken off patent and become far cheaper than the newer drugs, should cost-effectiveness play a role in the physician’s decision? Our role as clinicians is to give the most efficacious treatment to our patients, and clinical data based on rigorous trials should help us in making the right decisions. Pharmacogenomics is an important tool in optimizing health care, but like all tools it should be used appropriately.

## Figures and Tables

**Table 1:** **European Society of Cardiology (ESC) Unstable Angina/Non-ST-Elevation Myocardial Infarction (UA/NSTEMI) recommendations.**

**Recommendation**	**Class**	**Level**
Aspirin given at an initial loading dose of 150–300 mg and at a maintenance dose of 75–100 mg qd indefinitely	I	A
Ticagrelor (180-mg loading dose, 90 mg twice daily) for all pts at moderate-to-high risk of ischemic events regardless of initial treatment strategy and including those pretreated with clopidogrel (discontinued when ticagrelor is initiated)	I	B
Prasugrel (60-mg loading dose, 10-mg daily dose) is recommended for P2Y12 inhibitor-naïve pts in whom coronary anatomy is known and who are proceeding to PCI unless there is high risk of life-threatening bleeding	I	B
Clopidogrel (300-mg loading dose, 75-mg daily dose) for pts who cannot receive ticagrelor or prasugrel	I	A
A 600-mg loading dose of clopidogrel for pts scheduled for an invasive strategy when ticagrelor or prasugrel is not an option	I	B
A higher maintenance dose of clopidogrel 150 mg qd should be considered for the first 7 days in pts managed with PCI and without increased risk of bleeding	IIa	B
Increasing the maintenance dose of clopidogrel based on platelet function testing is not advised as routine, but may be considered in selected cases	IIa	B
Genotyping and/or platelet function testing may be considered in selected cases when clopidogrel is used	IIa	B

## Abbreviations:

ACC: American College of Cardiology
ACCF: American College of Cardiology Foundation
AHA: American Heart Association
CVD: cardiovascular disease
CYP: cytochrome P450
FDA: Food and Drug Administration
GRAVITAS: Gauging Responsiveness with A Verify Now assay-Impact on Thrombosis And Safety
PCI: percutaneous coronary interventions
PD: pharmacodynamic
PK: pharmacokinetic
